# Beyond the *p* factor: Is there a *d* factor?

**DOI:** 10.1002/jcv2.12051

**Published:** 2021-12-04

**Authors:** Samuele Cortese, Gonzalo Arrondo, Christoph U. Correll, Marco Solmi

**Affiliations:** ^1^ Centre for Innovation in Mental Health School of Psychology Faculty of Environmental and Life Sciences University of Southampton Southampton UK; ^2^ Clinical and Experimental Sciences (CNS and Psychiatry) Faculty of Medicine University of Southampton Southampton UK; ^3^ Solent NHS Trust Southampton UK; ^4^ Division of Psychiatry and Applied Psychology School of Medicine University of Nottingham Nottingham UK; ^5^ Hassenfeld Children's Hospital at NYU Langone New York University Child Study Center New York City New York USA; ^6^ Mind‐Brain Group Institute for Culture and Society University of Navarra Pamplona Spain; ^7^ Division of Psychiatry Research The Zucker Hillside Hospital Northwell Health New York New York USA; ^8^ Department of Psychiatry and Molecular Medicine The Donald and Barbara Zucker School of Medicine at Hofstra/Northwell New York New York USA; ^9^ Department of Child and Adolescent Psychiatry Charité Universitäts medizin Berlin Germany; ^10^ Department of Psychiatry University of Ottawa Ottawa Ontario Canada; ^11^ Department of Mental Health The Ottawa Hospital Ottawa Ontario Canada

“The mind and the body are not separate units, but one integrated system” Bernie Siegel, The Mind‐Body Connection (Siegel, 2019)

The current special issue of *JCPP Advances* focuses on the links between mental and physical conditions in children and adolescents. This broad topic is gaining traction in our field. Indeed, in the past years there has been an increasing awareness that disorders classically thought to be Central Nervous System conditions are actually associated with increased risk of alterations in other physiological systems (Qureshi & Mehler, 2013).

The studies published in the special issue contribute to further our understanding on the links between Attention‐Deficit/Hyperactivity Disorder (ADHD) and asthma (Sun et al., 2021); the relationship between anorexia nervosa and inflammatory bowel diseases (Larsen et al., 2021); the association of retinal vascular calibre with regional brain structure in adolescent bipolar disorder (Mio et al., 2021); the role of integrated care for child and adolescent health (Fazel et al., 2021); the acceptability, feasibility and impact of a drop‐in centre to address emotional and behavioural problems in children with chronic physical conditions (Bennett et al., 2021); the efficacy of group‐based Acceptance and Commitment Therapy for adolescents with multiple functional somatic syndromes (Kallesøe et al., 2021); and the evidence on physical exercise interventions in child and adolescent mental health (Carney & Firth, 2021). These studies add to and complement, with more fine‐grained information, an increasing body of empirical evidence on the relationships between mental disorders and physical conditions in children and adolescents. Indeed, for some associations in adults or in children/adolescents (e.g., ADHD and asthma [Cortese et al., 2018], ADHD and obesity [Cortese et al., 2016], depression and diabetes [Graham et al., 2020]) meta‐analytic synthesis is available. We are therefore at a point now where it is possible to provide high‐level, quantitative evidence synthesis of this body of evidence. In this regard, our group is conducting a large‐scale umbrella review of systematic reviews/meta‐analyses of studies on the association between mental and physical conditions. The project is referred to as AMASU (Association between Mental And Somatic conditions: an Umbrella review) and includes an adult as well as a paediatric section (Cortese et al., 2020). Ultimately, the projects’ aims are twofold: (1) to grade the strength of the evidence between specific mental disorders and specific physical conditions, using the classes of evidence proposed by Ioannidis’ group (Tsilidis et al., 2015) (class I: convincing; class II: highly suggestive; class III: suggestive; class IV: weak); (2) based on the TRANSD recommendations by Fusar‐Poli et al. (2019), to ascertain to which extent specific mental disorders are selectively associated with specific somatic conditions or if there are transdiagnostic, across‐spectra, or diagnostic spectrum‐specific associations between mental disorders and somatic conditions. AMASU will be complemented by AMASU EXTENSION, providing a series of meta‐analyses on associations for which primary studies, but not meta‐analyses, are available yet. Should the result of AMASU point to convincing transdiagnostic associations between mental and physical conditions, they would provide a compelling rationale for a line of research aimed at establishing if there is a common factor that accounts for both psychopathology and physical disease.

Many readers will be familiar with the so called ‘*p* factor’ in psychopathology, which is grounded on the finding that psychopathological disorders are correlated not only at the disorder level, but also at the spectra level. Indeed, the correlation between externalising and internalising spectra has been estimated at ∼0.5, and the correlation between internalising and thought disorders at ∼0.6 (Wright et al., 2013). Based on these findings, Lahey et al. (2012) suggested that there may be one single underlying factor that accounts for an individuals’ propensity to develop any and every common psychopathological disorder. In other words, most common psychiatric disorders are unified by a single psychopathology dimension. Subsequently, Caspi et al. (2014) proposed that, just as there is a general factor (*g* factor) of cognitive abilities that accounts for the positive correlation among scores on the subtests of intelligence, there may be a general factor (‘*p*’) of psychopathology. Caspi et al. (2014) tested and confirmed this hypothesis using data from the Dunedin Multidisciplinary Health and Development Study. Considering dimensionality, persistence, co‐occurrence, and sequential comorbidity of mental disorders across the age span from adolescence to midlife, they found that mental disorders were explained by three higher‐order factors (Internalizing, Externalizing, and Thought Disorder) but were explained even better by one single General Psychopathology dimension, the *p* factor.

Now, the question is: is there a *d* (= disease) factor underlying the propensity to mental as well as physical diseases? Future research will be able to tell. Should this putative *d* factor be confirmed, it would have substantial implications not only from a theoretical or even philosophical standpoint, but also from a practical perspective, providing a strong rationale for the integrated care for children and adolescents advocated in this issue by Fazel et al. (2021) and Bennett et al. (2021).

Therefore, this special issue of *JCPP Advances* projects us in an important future development of our discipline. We look forward to growing, multidisciplinary high‐quality work in this area that has the potential to elucidate underlying pathophysiological dimensions that are hoped to inform novel forms of interventions to benefit the body and the mind in people with mental and/or physical disorders.

## CONFLICT OF INTERESTS

Samuele Cortese is Deputy Editor of the *Journal of the American Academy of Child and Adolescent Psychiatry*, *Deputy Editor of Evidence Based Mental Health*, and Associate Editor of Child and Adolescent Mental Health. He is also a member of the Editorial Advisory Board for JCPP *Advances*. Christoph U. Correll is also a member of the Editorial Advisory Board for JCPP *Advances*. Marco Solmi is Joint Editor of *JCPP Advances*. The remaining authors have declared that they have no competing or potential conflicts of interest. [Corrections made on 22 June 2022, after first online publication: This Conflict of Interests statement has been updated in this version.]
